# Knowledge is Power: Rethinking Healthcare for Culturally and Linguistically Diverse Patients

**DOI:** 10.7759/cureus.45712

**Published:** 2023-09-21

**Authors:** Elissa M Monteiro, Carly Hyde, Dilian Guardado, Kashia A Rosenau, Alice Kuo

**Affiliations:** 1 School of Education/Department of School Psychology, University of California Riverside, Riverside, USA; 2 Psychiatry, University of California Riverside School of Medicine, Riverside, USA; 3 Fielding School of Public Health, University of California Los Angeles, Los Angeles, USA; 4 Division of Medicine-Pediatrics, University of California Los Angeles David Geffen School of Medicine, Los Angeles, USA

**Keywords:** access to healthcare and health outcomes of vulnerable populations, culturally and linguistically diverse, special education, autism, equity and inclusion

## Abstract

Autism is a developmental disability that exists across racial, ethnic, linguistic, and socioeconomic boundaries. Unfortunately, the lived experiences of autistic individuals and their families as supported by evidence in the existing literature suggest that culturally and linguistically diverse families’ engagement in healthcare and education face a multitude of challenges, particularly during high-stakes meetings and healthcare appointments (e.g., Individualized Education Plan meetings, patient visits, and diagnostic results interpretation meetings). These challenges prevent culturally and linguistically diverse autistic individuals from accessing adequate care. In this paper, we propose solutions to be adopted by healthcare and education systems to address those challenges. First, we urge providers to address the systemic problems that commonly occur during meetings. Second, we propose service providers adopt a cultural and linguistic ‘match’ process. We recommend asking families about their specific language preferences and ensuring the selection of translators who speak the family’s preferred language and dialect. Employing these transformations will require education and healthcare systems to allocate more resources for translation services to enhance the training and recruitment of interpreters and ensure that interpreter-family pairs are provided time for consultation prior to high-stakes meetings. Ultimately, these adaptations to the service provision environment would produce opportunities for translators to act as cultural liaisons and, with time, become trusted partners for families.

## Editorial

Autism is a developmental disability that exists across racial, ethnic, linguistic, and socioeconomic boundaries. In the United States, one in five children are from culturally and linguistically diverse (CLD) backgrounds, meaning they have a caregiver who speaks one of the 350 non-English languages used nationally, and up to 10% of these caregivers speak limited English [1]. Non-English-speaking status increases the risk of poor access to quality medical care and intervention services within healthcare and education systems, lower rates of insurance coverage, reduced utilization of preventive services, delayed autism diagnosis, and fewer intervention service hours [2-8]. The consequences of inadequate health care for autistic CLD individuals are disparate rates of preventable diagnoses (e.g., mental health, sleep difficulties, and digestive problems), as well as higher long-term health care costs, higher rates of hospitalization, lower quality of life, and shorter life-expectancy when compared to children with autism whose families are primarily English speaking [9-12].

There are laws such as the Patient Protection and Affordable Care Act (ACA) that prohibit the discrimination of patients by medical professionals [13]. Laws like these are designed to promote family engagement in healthcare and education. A multitude of challenges during high-stakes meetings and appointments (e.g., Individualized Education Plan meetings, patient visits, diagnostic results interpretation meetings) prevent autistic CLD children from accessing adequate care. These meetings are intended to provide a space to inform caregivers of special considerations to promote children’s well-being, set goals for their academic success or health, and address parents’ concerns. For CLD families, these goals can be especially difficult to achieve. Providers may inaccurately identify the family’s preferred language or dialect, may misunderstand non-verbal communication, or use unfamiliar terminology that trained interpreters are unprepared to describe [14]. This miscommunication results in strained relationships, loss of vital information, and poor understanding of a child’s coordinated care plan [3,15,16]. Parents may be unfamiliar with their rights within the complex systems and may feel uncomfortable asking questions due to inherent power dynamics. For example, there may be cultural differences in the degree of deference to medical professionals in a specialized setting such as a medical clinic. Educational materials may not be available in the family’s preferred language or may not match their level of health literacy [9,17]. Cultural differences in beliefs regarding autism and child developmental milestones can deter parents from collaborating with service providers, and providers may not take sufficient time to understand the needs of a family or adapt their strategies to the family's strengths and preferences [9]. This can lead to caregivers feeling undervalued and unheard, which can be a precursor for the discontinuation of services or poor adherence to interventions [18]. This discrepancy between patients’ and service professionals’ identities engenders tension in service provision with the patients who may need it most.

To address these challenges experienced by CLD children, we propose two complementary solutions for school-based and healthcare service providers. First, providers must address the systemic problems that commonly occur during meetings. This includes asking families about their specific language preferences and ensuring the selection of translators who speak the family’s preferred language and dialect in advance of the scheduled meeting [14]. Despite extensive training, interpreters might not be familiar with the vocabulary related to special education and neurodevelopmental disabilities often used by providers [3]. Interpreters should be provided a glossary of terms to aid them in appropriately describing complex jargon. Interpreters should also communicate with providers about nuances in non-verbal communication and cultural norms to facilitate respectful interactions. Interpreters should act as a voice, not as a filter for communication. This means they should translate the professional’s statements word for word. Therefore, in preparation for work with autistic populations, interpreters should be made aware of characteristics of autism (e.g., expressive and receptive language differences, and changes in vocal intonation). In preparation for working with individual families, interpreters should be aware of whether these characteristics of autism overlap with cultural norms (e.g., eye contact). We urge interpreters to hold both aspects of the individual they are translating for in mind, and we encourage interpreters to directly translate for the individual rather than provide corrections to their language. Finally, providers should make every effort to provide written summaries of meetings in the family’s preferred language within a reasonable timeframe.

Second, a cultural and linguistic ‘match’ process should happen at the beginning of the referral process. We argue that building rapport between the interpreter and the patient's family under low-stakes conditions would establish familiarity and, thus, increase trust between the patient and interpreter. This would require service providers to coordinate a time and space for the translator and family to meet before provider appointments. During the meeting, translators should inquire about the family’s treatment priorities and concerns, further encouraging caregivers to reflect on their own goals and prepare to discuss their needs with providers. Establishing a familiar relationship can facilitate parents feeling seen and cared for, an aspect often missing from these meetings [2,4].

Employing this practice will require education and healthcare systems to allocate more resources for translation services to enhance the training and recruitment of interpreters and ensure that interpreter-family pairs are provided time for consultation prior to high-stakes meetings. Once assigned, these pairs should remain consistent to maximize positive relationships and outcomes while minimizing power imbalances. Given that many minority families respond positively to peer relationships, healthcare and education systems should consider hiring and training local community members [7]. It can be challenging for providers and educators to maintain consistency between meetings, with staff often attending only a portion of the meeting to accommodate scheduling. A consistent translator can offer stability, especially during contentious meetings. Both healthcare and education systems should consider how to responsibly leverage this relationship while ensuring that families receive appropriate guidance and expertise.

The consequences of inadequate CLD for autistic individuals include higher rates of hospitalization, lower quality of life, and shorter life expectancy compared to their English-speaking counterparts [9-12]. Given the variety of co-occurring mental and physical health conditions associated with autism and considering the risks that CLD autistic individuals experience within healthcare and education systems, it is essential that we accommodate healthcare systems to protect this population from harm [2-8]. Ultimately, adaptations to the service provision environment, such as the proposed adaptations described in this editorial, would provide opportunities for translators to build rapport with families. While these solutions may require direct monitoring and active flexibility to implement, improving the quality of translation services for CLD families is critical to fostering a collaborative, supportive, and respectful environment for families and is necessary to improve the quality of services provided in healthcare and education settings.

## References

[1] Angell AM, Empey A, Zuckerman KE Chapter four - a review of diagnosis and service disparities among children with autism from racial and ethnic minority groups in the United States. International Review of Research in Developmental Disabilities.

[2] Timmins CL (2002). The impact of language barriers on the health care of Latinos in the United States: a review of the literature and guidelines for practice. J Midwifery Womens Health.

[3] Lim N, O’Reilly M, Sigafoos J, Lancioni GE, Sanchez NJ (2021). A review of barriers experienced by immigrant parents of children with autism when accessing services. Rev J Autism Dev Disord.

[4] St Amant HG, Schrager SM, Peña-Ricardo C, Williams ME, Vanderbilt DL (2018). Language barriers impact access to services for children with autism spectrum disorders. J Autism Dev Disord.

[5] Zuckerman KE, Mattox K, Donelan K, Batbayar O, Baghaee A, Bethell C (2013). Pediatrician identification of Latino children at risk for autism spectrum disorder. Pediatrics.

[6] Luelmo P, Sandoval Y, Kasari C (2022). Undocumented Mexican mothers of children with autism: navigating the health care and educational service systems. Int J Dev Disabil.

[7] Locke J, Kang-Yi CD, Pellecchia M, Marcus S, Hadley T, Mandell DS (2017). Ethnic disparities in school-based behavioral health service use for children with psychiatric disorders. J Sch Health.

[8] Lopez K, Magaña S, Morales M, Iland E (2019). Parents taking action: reducing disparities through a culturally informed intervention for Latinx parents of children with autism. J Ethn Cult Divers Soc Work.

[9] Croen LA, Zerbo O, Qian Y, Massolo ML, Rich S, Sidney S, Kripke C (2015). The health status of adults on the autism spectrum. Autism.

[10] Frazier TW, Dawson G, Murray D, Shih A, Sachs JS, Geiger A (2018). Brief report: a survey of autism research priorities across a diverse community of stakeholders. J Autism Dev Disord.

[11] Van Cleave J, Holifield C, Neumeyer AM, Perrin JM, Powers E, Van L, Kuhlthau KA (2018). Expanding the capacity of primary care to treat co-morbidities in children with autism spectrum disorder. J Autism Dev Disord.

[12] Bishop-Fitzpatrick L, Kind AJ (2017). A scoping review of health disparities in autism spectrum disorder. J Autism Dev Disord.

[13] (2019). H.R.1425 - Patient Protection and Affordable Care Enhancement Act. Patient Protection and Affordable Care Act of 2019, Pub. L. No. 111-148, 124 Stat. 119.

[14] Rossetti Z, Sauer JS, Bui O, Ou S (2017). Developing collaborative partnerships with culturally and linguistically diverse families during the IEP process. Teach Except Child.

[15] Allen MP, Johnson RE, McClave EZ, Alvarado-Little W (2020). Language, interpretation, and translation: a clarification and reference checklist in service of health literacy and cultural respect. NAM Perspect.

[16] Papoudi D, Jørgensen CR, Guldberg K, Meadan H (2021). Perceptions, experiences, and needs of parents of culturally and linguistically diverse children with autism: a scoping review. Rev J Autism Dev Disord.

[17] García SB, Méndez Pérez A, Ortiz AA (2000). Mexican American mothers’ beliefs about disabilities: implications for early childhood intervention. Remedial Spec Educ.

[18] Stahmer AC, Vejnoska S, Iadarola S (2019). Caregiver voices: cross-cultural input on improving access to autism services. J Racial Ethn Health Disparities.

